# Supplementary figures and images for: The impact of tinnitus on Dutch general practices: A retrospective study using routine healthcare data
Source: PLoS One. 2024 Nov 15;19(11):e0313630. doi: 10.1371/journal.pone.0313630 (PMC11567631; doi:10.1371/journal.pone.0313630)

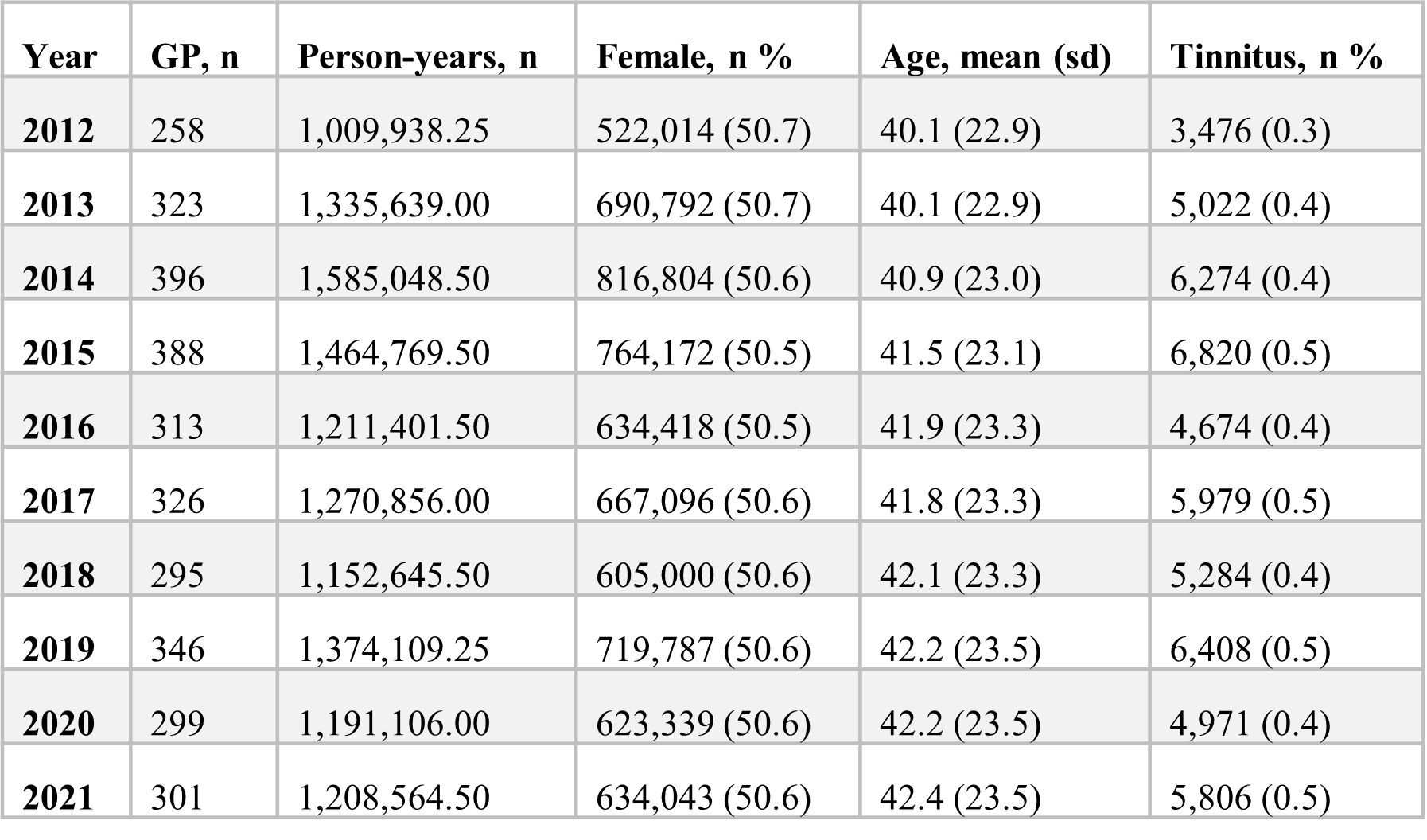

Supplement: S1 Table — GP = general practice. sd = standard deviation. (TIF) [file pone.0313630.s001.tif]

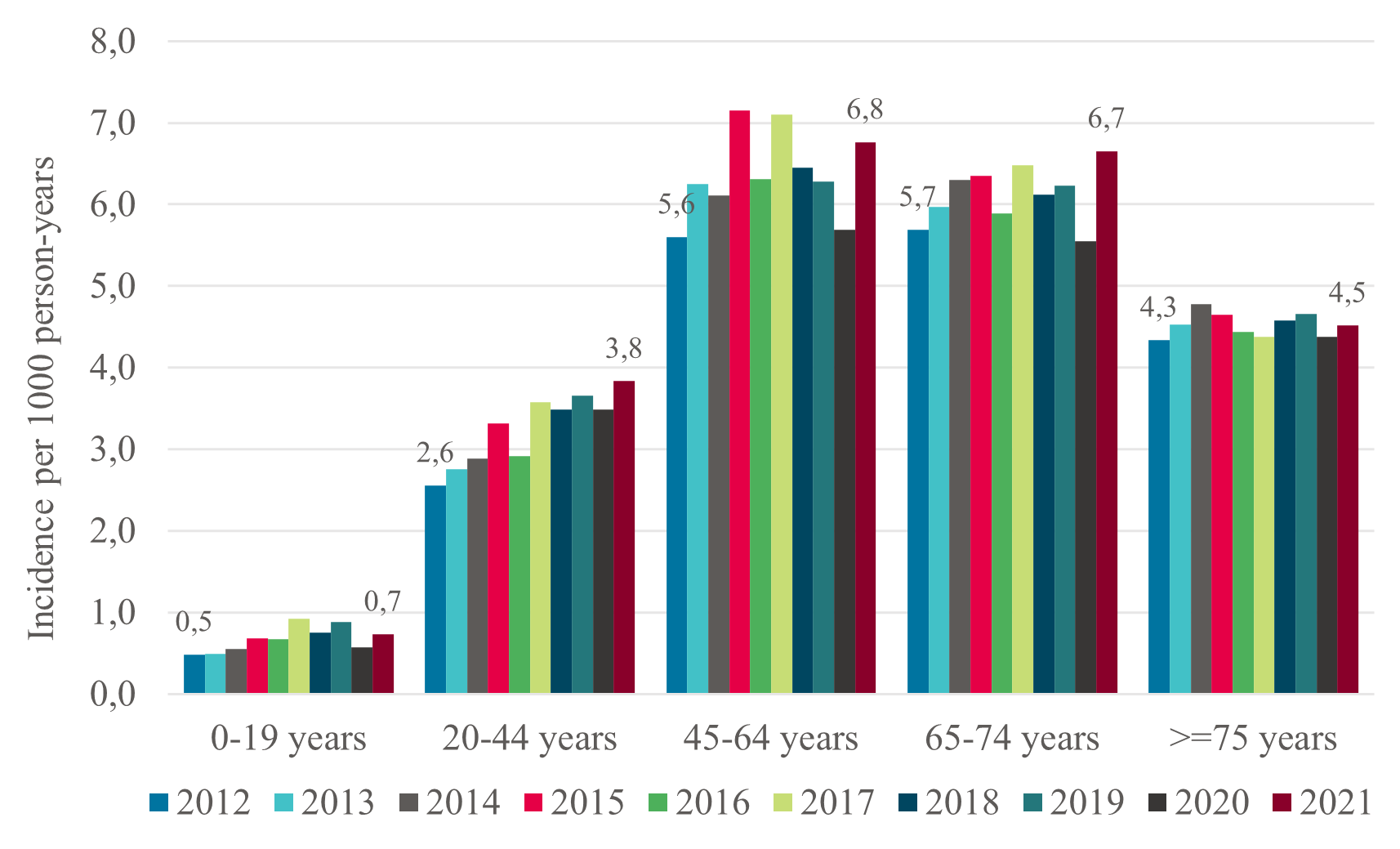

Supplement: S1 Fig — Incidence: the number of new diagnoses divided by total person-years, per 1000 person-years. (TIF) [file pone.0313630.s002.tif]

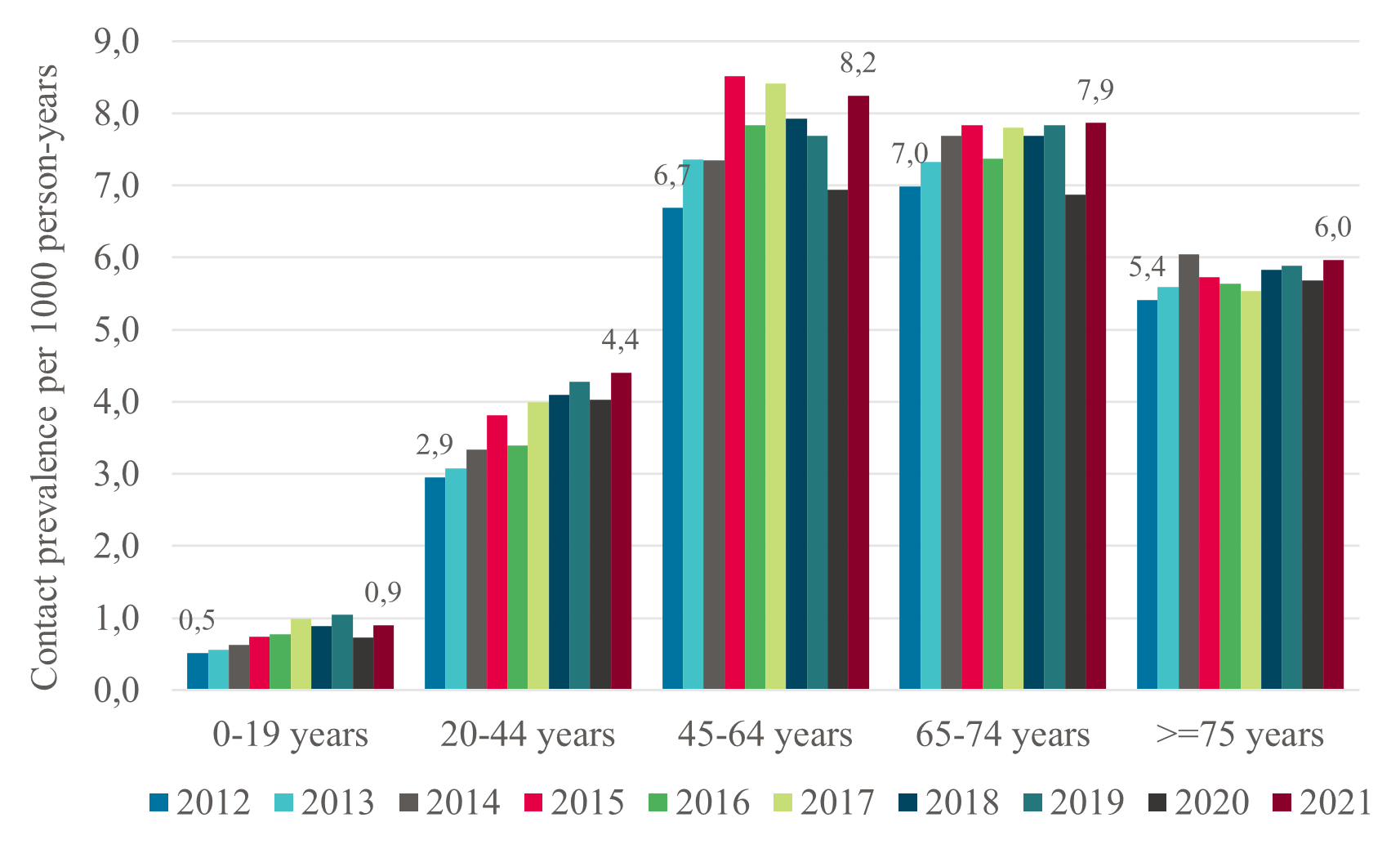

Supplement: S2 Fig — Contact prevalence: the number of patients with a general practice contact for tinnitus divided by total person-years, per 1000 person-years. (TIF) [file pone.0313630.s003.tif]

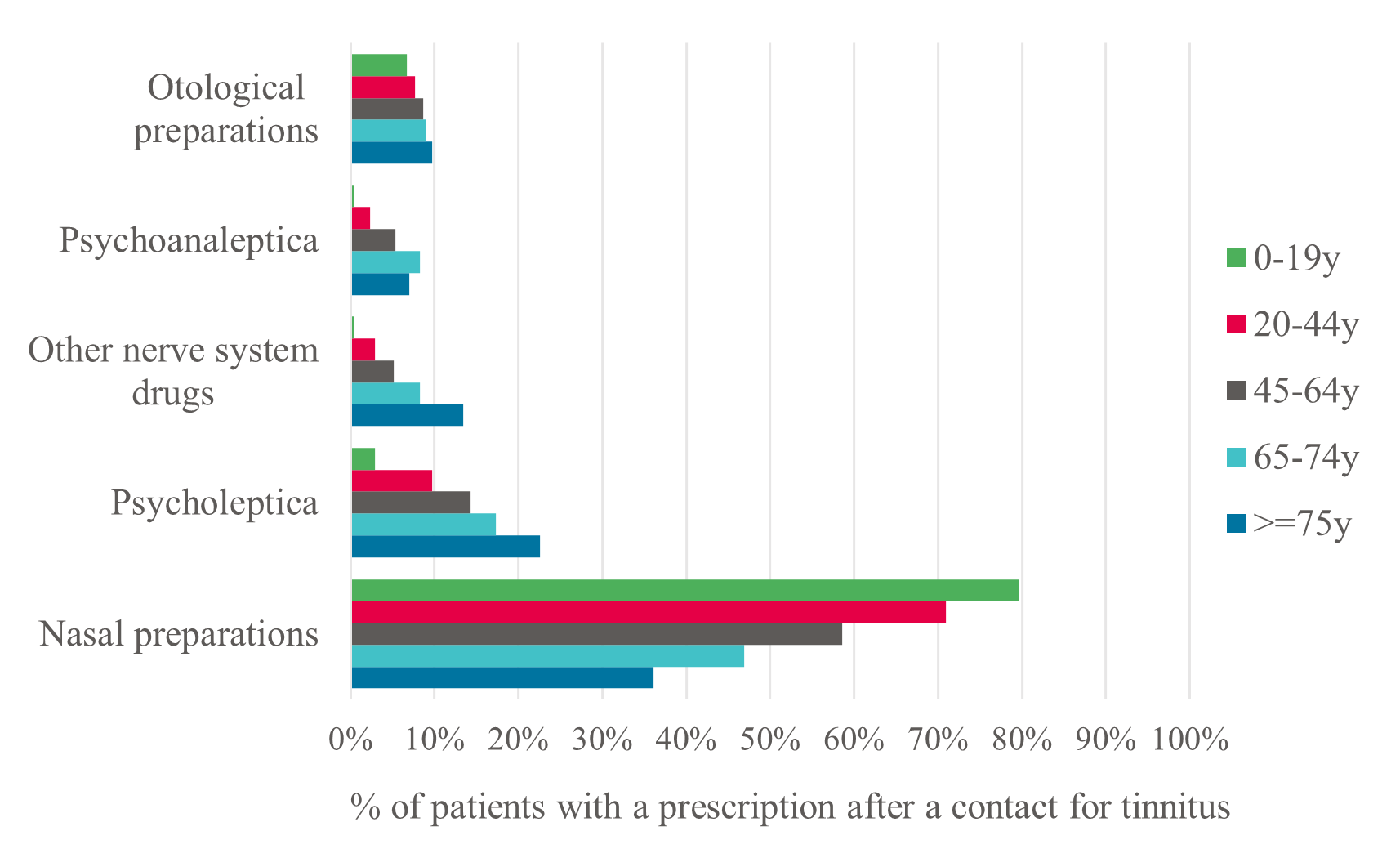

Supplement: S3 Fig — (TIF) [file pone.0313630.s004.tif]

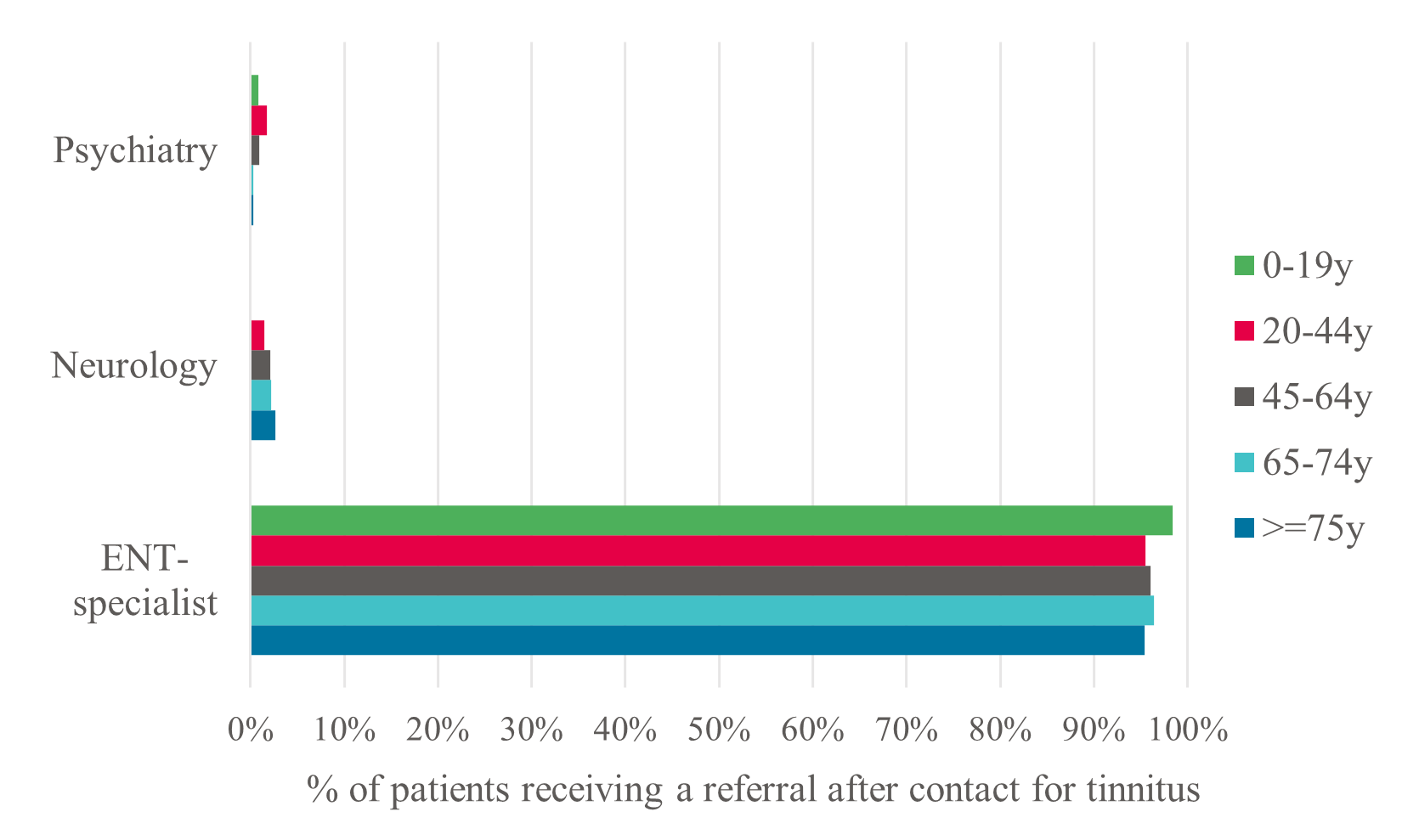

Supplement: S4 Fig — ENT = ear, nose and throat. (TIF) [file pone.0313630.s005.tif]
